# Investigation of Poling for Pb(Zr, Ti)O_3_/Pb(Zr, Ti)O_3_ Sol–Gel Composite †

**DOI:** 10.3390/mi16070760

**Published:** 2025-06-28

**Authors:** Mako Nakamura, Ryota Ono, Makiko Kobayashi

**Affiliations:** 1 Graduate School of Science and Technology, Kumamoto University, Kumamoto 860-8555, Japan; 242d9364@st.kumamoto-u.ac.jp (M.N.);; 2 Faculty of Advanced Science and Technology, Kumamoto University, Kumamoto 860-8555, Japan

**Keywords:** DC poling, sol–gel composite, ultrasonic transducers, phased-array

## Abstract

Phased-array ultrasonic transducers using sol–gel composites face challenges in terms of polarization uniformity when using conventional corona poling. Pb(Zr, Ti)O_3_ (PZT)/PZT composites with a thickness of 25 µm were fabricated on 3 mm thick titanium substrates, and the samples were poled by AC poling, DC poling, and corona discharge poling at RT. It was found that the polarization direction could be controlled by the voltage off-phase angle. When poling was performed with a voltage off-phase angle of 90°, applied voltage of 200 V (rms), 10 cycles, and frequency of 1 Hz, average values and standards of measured piezoelectric constant *d*_33_ of −35.1 ± 0.8 pC/N and ultrasonic sensitivity of 11.4 ± 0.1 dB were obtained. Furthermore, the AC-poled samples demonstrated smaller variations in *d*_33_ and ultrasonic sensitivity compared with the corona-poled samples, and higher values of *d*_33_ and ultrasonic sensitivity compared with the DC-poled samples, indicating the potential of AC poling for PZT/PZT sol–gel composites with large areas.

## 1. Introduction

Phased-array ultrasonic transducers are extensively used in medical diagnostics and industrial testing. Phased-array technology electronically regulates the deflection and focus of ultrasonic beams by controlling the phase of the ultrasonic waves transmitted and received by multiple ultrasonic transducers within the flaw detector, thereby enabling the detection of defects that are challenging to identify using conventional ultrasonic transducers. With advances in phased-array technology, the demand for high-performance transducers increases. For instance, the flexibility of transducers facilitates the ultrasonic nondestructive inspection of complex shapes [1,2]. Furthermore, the enhanced transducer heat resistance enables defect detection at elevated temperatures (previously unfeasible); accordingly, defect detection is extensively used in industrial applications [3,4].

The sol–gel composite method is used to fabricate porous piezoelectric films by spraying a mixture of piezoelectric powder and sol–gel solution onto a substrate [5]. The fabrication method was based on a sol–gel method. The mixing ratio of the powder to the sol–gel solution is an important parameter in the fabrication process; however, the mixing ratio of the final film changes depending on the parameters at the time of spraying, and the exact mixing ratio is unknown. Nevertheless, the ratio of the sol–gel phase to the powder phase was estimated to be very small because it was not reflected in the X-ray diffraction (XRD) pattern after film formation. A film thickness of approximately 5–20 µm can be achieved in a single spraying process [5]. The porosity can be controlled by the fabrication parameters, and was fabricated between 10 and 30%, which is suitable for ultrasonic device applications, and approximately 20% for the parameters used in this experiment. The resulting porosity confers physical flexibility [6] and heat resistance [7] without the need for a backing material or a couplant. Moreover, the porous structure contributes to the reduction in crosstalk between the elements, and the kerfless array method [8,9,10] can be employed to eliminate the costly dicing process, thereby enabling the creation of cost-effective phased-array transducers. Ultrasonic phased arrays utilizing sol–gel composites have been developed in recent years [11]. For further performance enhancement of ultrasonic phased arrays utilizing sol–gel composites, a substantial number of elements must exhibit equivalent ultrasonic characteristics, necessitating a high uniformity of the film quality and degree of polarization. DC poling, in which a DC voltage is applied to a sample in an oil bath, is employed for conventional bulk piezoelectric ceramics. For the poling of porous sol–gel composites, corona discharge poling is utilized to mitigate contamination by the oil bath, which inhibits dielectric breakdown [6,7]. In corona discharge poling, a high electric field is applied between the needle and flat plate electrodes to generate a corona discharge. When an insulator is placed between these electrodes, ions move from the needle electrode to the flat plate electrode during discharge and impinge on the surface of the insulator, charging the surface and creating a strong electric-field gradient. As corona discharge poling does not require an oil bath, it is primarily employed to polarize porous piezoelectric polymers [12,13,14] and can apply a high electric field without inducing a dielectric breakdown [15,16]. However, corona discharge poling presents challenges in uniformly polarizing the area required for the numerous electrodes used in phased-array ultrasonic transducers, necessitating a method that can uniformly polarize a large area.

AC poling is a polarization technique that uses AC electric fields instead of DC electric fields [17,18,19,20,21,22,23,24,25]. AC poling on Pb(Mg_1/3_Nb_2/3_)O_3_ (PMN)-PbTiO_3_ (PT) and Pb(In_1/2_Nb_1/2_)O_3_ (PIN)-PMN-PT improves their piezoelectric properties [17,18,19,20]. J. Xu et al. reported AC poling of PMN-0.25PT single crystals with a 40% increase in *d*_33_ and denser domain walls [17]. Y. Yamashita et al. reported that AC poling of PMN-Pb(Zr, Ti)O_3_ (PZT) single crystals fabricated by the solid-phase crystal growth method produced a unique 109° domain wall with the highest properties [21]. J. Xiong et al. concluded that the formation of uniform domain patterns by alternating polarization is an important factor in improving the piezoelectric properties [22]. Many studies have focused on the changes in the domain structure of ferroelectric single crystals due to AC poling, but there have also been reports on ferroelectric ceramics. Kim et al. reported that in lead-free Bi-based ceramics, applying AC poling before DC poling increased the degree of domain orientation and *d*_33_ [23]. In the case of the AC polarization of BaTiO_3_ (BT) ceramics, an improvement in *d*_33_ has been reported owing to a reduction in the domain size [24]. Ma et al. observed improved piezoelectric properties and denser domain walls when AC poling was applied to PZT-5H ceramics [25]. While there have been reports on AC poling for a variety of ferroelectric materials, there have been no reports on PZT/PZT sol–gel composites. Because the capacitance and resistance are low in sol–gel composites owing to their porous nature, polarization at low-frequency AC may result in better properties because the maximum transient current is lower than that of DC, thus suppressing dielectric breakdown and microcracking. Thus, the potential of AC poling in PZT/PZT and Bi_4_Ti_13_O_12_ (BiT)/PZT [26] was evaluated using *d*_33_ values. In this study, to clarify the effectiveness of AC poling on sol–gel composites, only PZT/PZT sol–gel composite samples were evaluated comprehensively based on *d*_33_, relative dielectric constant, dielectric loss, and ultrasonic measurement results. After the AC poling condition optimization, PZT/PZT samples were poled by AC poling, DC poling, and corona discharge poling, and the abovementioned electric, piezoelectric, and ultrasonic properties were compared.

## 2. Materials and Methods

To prepare the sol–gel composite film, the self-made PZT sol–gel and PZT powder (HIZIRCO L, Hayashi Chemical Industry Co., Shiga, Japan) were mixed for 24 h using a ball-milling machine. This process yielded a mixture suitable for spraying. In this experiment, PZT powder and PZT sol–gel with different material properties were utilized, as listed in Table 1. In Ref. [27], the sol-gel-derived PZT thin film was deposited on a silicon substrate with a Ti/Pt bottom electrode and a Cr/Au top electrode. The film thickness was approximately 1.13 µm.

**Figure 1:**
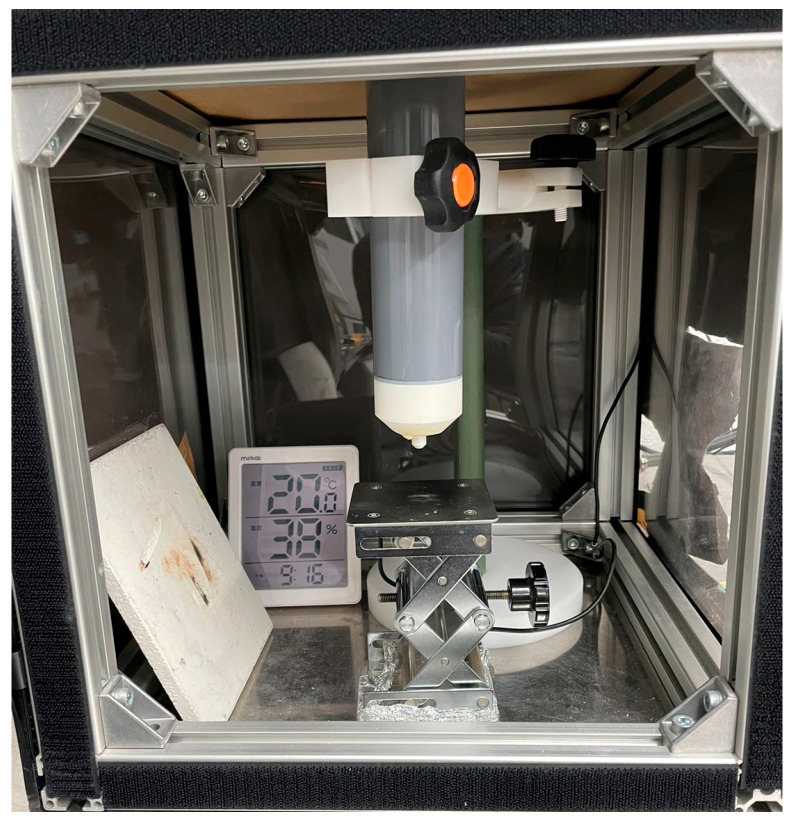
Optical image of the corona discharge poling process.

**Figure 2:**
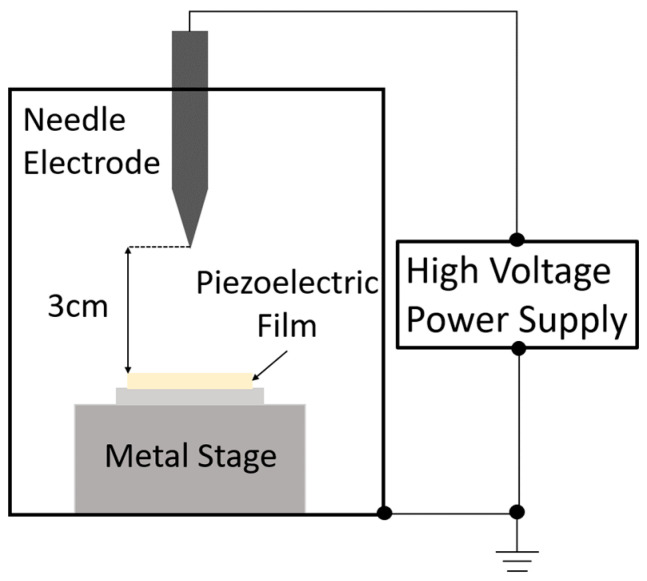
Schematic of the corona discharge poling process.

The prepared mixture was applied to a titanium substrate (30 mm square, thickness of 3 mm) using an automatic sprayer. Titanium is one of the most common substrates used to prepare sol–gel composites and exhibits excellent heat resistance. Subsequently, the samples were dried at 150 °C for 10 min and sintered at 650 °C for 5 min each. These processes were repeated until the target film thickness (25 μm) was reached. To prevent damage to the top electrode by ozone and other plasma products, corona discharge poling was performed before fabricating the top electrode. The apparatus for corona discharge poling is shown in Figure 1. A schematic representation is presented in Figure 2.

A DC voltage of approximately −40 kV was applied to the sol–gel composite before preparing the top electrode, with the distance to the needle electrode set at 3 cm and the humidity maintained at values < 20%. As corona discharge poling is conducted in a high-humidity environment, the electric field is not efficiently applied to the sol–gel composite [17].

**Figure 3:**
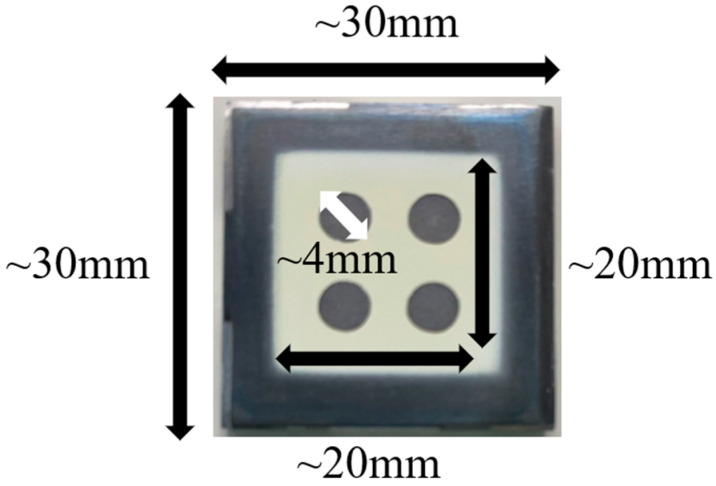
Typical optical image of a PZT/PZT sample with top electrodes on a titanium substrate of dimensions 30 mm × 30 mm × 3 mm.

The top electrode was fabricated by sputtering the sol–gel composite. The top electrode was composed of platinum and had a diameter of 4 mm. Optical images of the prepared PZT/PZT composite are shown in Figure 3.

**Figure 4:**
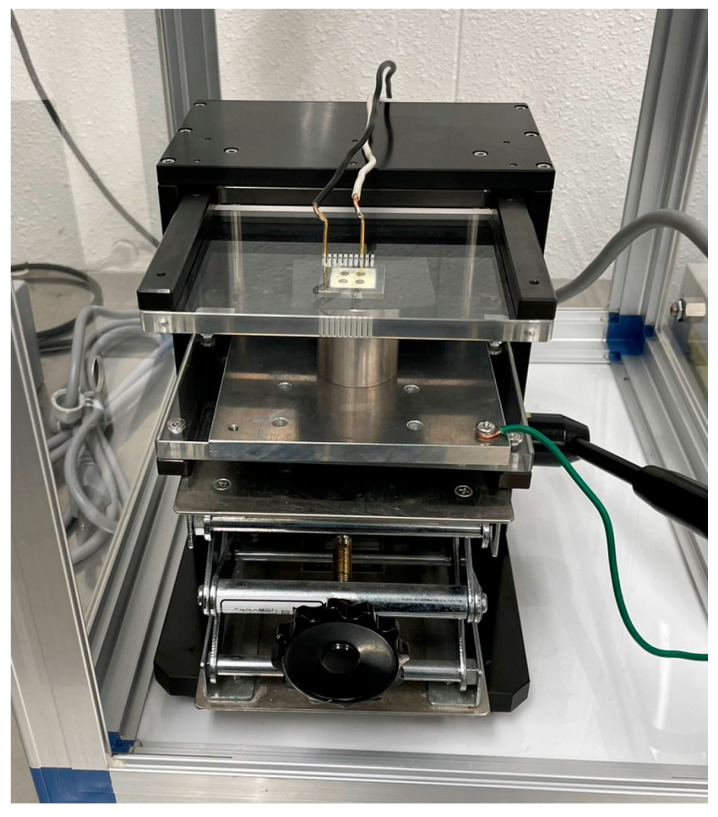
Optical image of the electrical contact system during the AC and DC poling processes.

**Figure 5:**
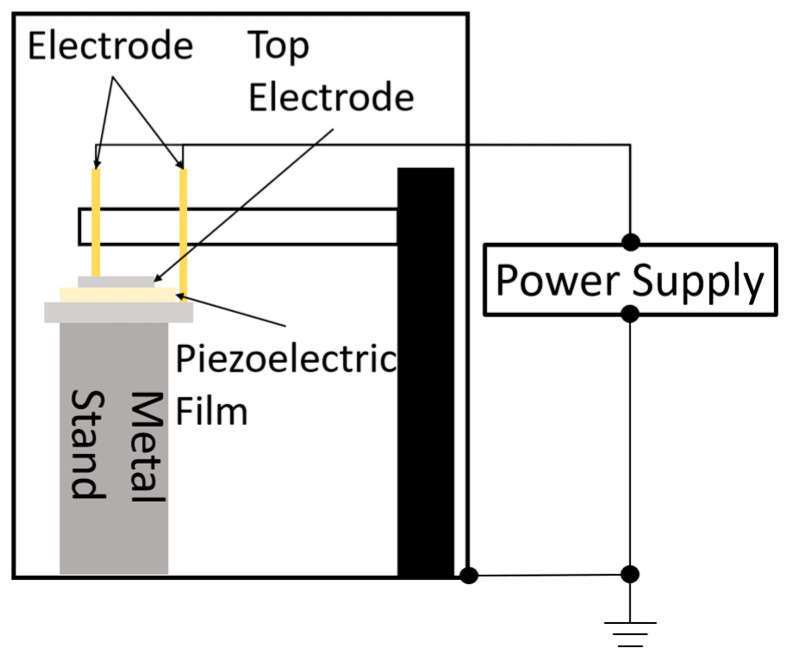
Schematic of the AC and DC poling processes.

After fabricating the upper electrode, DC and AC poling were performed. The electrical connection apparatus used in this experiment for AC and DC poling is shown in Figure 4. A schematic of the poling system is shown in Figure 5.

**Figure 6:**
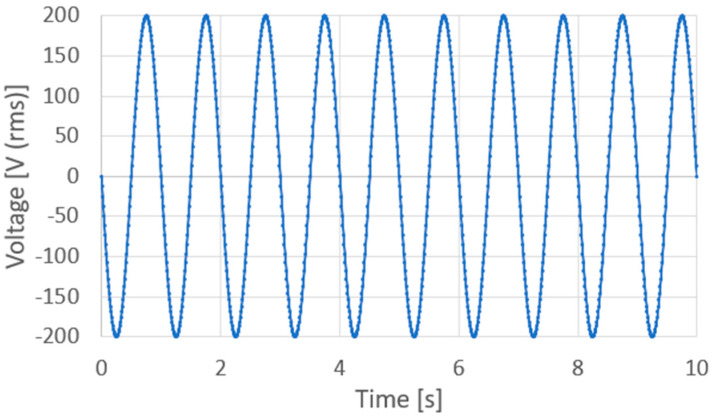
Voltage application chart for AC poling (1 Hz, 10 cycles or more).

**Figure 7:**
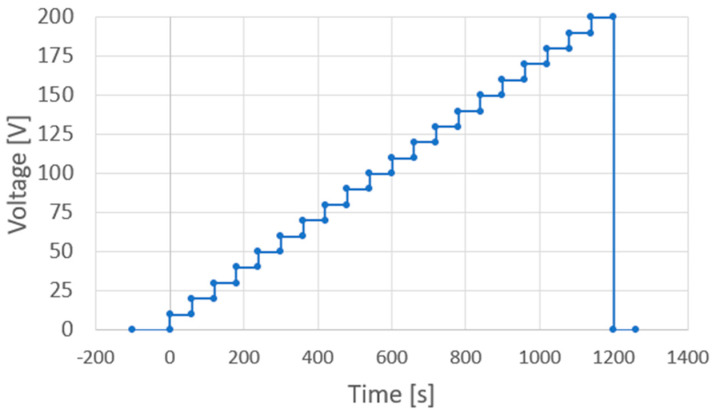
Voltage application chart for DC poling.

AC and DC voltages were applied using an AC/DC-stabilized power supply (PCR1000WEA, Kikusui Electronics Industry, Kanagawa, Japan). This voltage generated an electric field that was applied to the sol–gel composite via the probes and platinum upper electrode. During AC poling, an AC electric field was applied to control the applied voltage off-phase angle, applied voltage, number of applied cycles, and frequency, controlled via the front panel interface of the KIKUSUI PCR1000WEA system. It should be noted that the voltage off-phase angle refers to the specific phase position (in degrees) within the AC waveform at which the output voltage is turned off. This parameter allows for precise control of the voltage shutoff timing relative to the waveform. Figure 6 shows a voltage application chart where an AC voltage is applied with an applied voltage off-phase angle of 0°, an applied voltage of 200 V (rms), 10 application cycles, and a frequency of 1 Hz. In contrast, during DC polarization, the voltage was increased by 10 V every minute, and 200 V was applied at the end, as shown in the voltage application chart in Figure 7. To suppress dielectric breakdown in the porous sol–gel composite films, the maximum DC field was applied for one minute during the DC poling process.

## 3. Results and Discussion

### 3.1. AC Poling Condition Optimization for PZT/PZT

**Figure 8:**
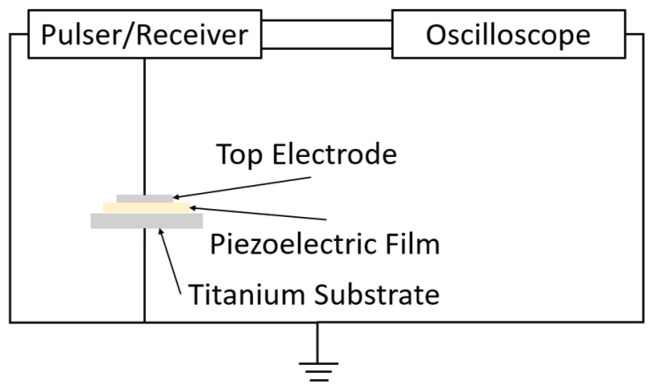
Experimental system for ultrasonic measurement.

After poling, the piezoelectric constant *d*_33_ was measured using a *d*_33_ meter (ZJ-3B, Institute of Acoustics, Chinese Academy of Sciences, Beijing, China), and the relative permittivity and dielectric loss were measured using an LCR meter (IM3523, Hioki E.E. Co., Ueda, Japan). The ultrasonic waves were measured using the pulse-echo method with a pulser/receiver (P/R) and an oscilloscope. The experimental system used for ultrasonic measurements is shown in Figure 8.

Following the ultrasonic measurements, the sensitivity was calculated using the following equation, where *V*_1_ represents the ideal amplitude (0.1 V), *V*_2_ denotes the first wave amplitude of the reflected wave, and *P*/*R Gain* signifies the gain of the P/R (DPR300, JSR).

(1) $$Sensiticity=-\left(20\log\frac{V_{1}}{V_{2}}\right)+P/RGain$$

Initially, the PZT/PZT was poled by varying the voltage off-phase angle, applied voltage, number of cycles, and frequency. Table 2 lists the measurement results for *d*_33_ as a function of the voltage off-phase angle of the voltage output. In this study, phase angles of 0°, 90°, 180°, and 270° were selected as they correspond to the peak or post-peak zero-crossing voltage levels, which reliably exceed the coercive field required for polarization switching. The values in Table 2 are the measured *d*_33_ values. The actual *d*_33_ is a positive value; however, negative values are also shown to indicate the polarization direction. In this study, negative values of *d*_33_ are consistently shown as they are. The data in Table 2 demonstrates that the direction of the AC poling is determined by the voltage off-phase angle. Although some variation in response was observed, it is attributed to sample-specific differences. The phase must be set to unity to stabilize the poling conditions. In this study, an angle of 90° was chosen to polarize in the negative direction. Because the poling direction changes when the threshold of this voltage off-phase angle is exceeded, it is important to consider the voltage off-phase angle, which indicates the timing to stop AC poling. The negative direction was selected because the P/R applied an electric field in this direction. Typically, P/R measures ultrasonic waves using a negative electric field to prevent damage to piezoelectric devices. In a previous study, we reported that a negative electric field applied by P/R caused transducers with positive corona discharge poling to be polarized in the negative direction, even though the extent of polarization was minimal [9].

**Figure 9:**
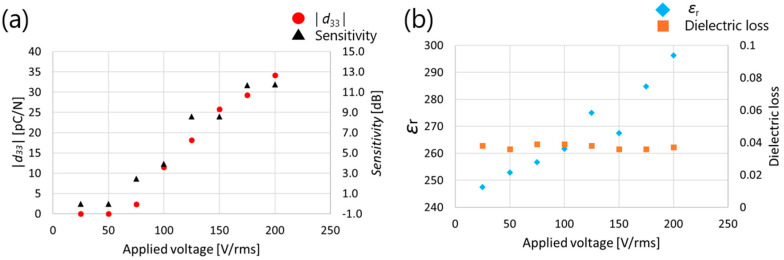
Measured (**a**) *d*_33_ (**b**) relative dielectric constant results for AC poling (60 cycles, 1 Hz) as a function of the applied voltage on PZT/PZT.

Figure 9 shows the measurement results of *d*_33_, ultrasonic sensitivity, relative permittivity, and dielectric loss with increasing applied AC voltage. In Figure 9, when 25 V (rms) and 50 V (rms) were applied, *d*_33_ and ultrasonic sensitivity values were 0, indicating that the threshold voltage for poling was not exceeded. Between 75 V (rms) and 200 V (rms), *d*_33_, ultrasonic sensitivity, and dielectric constant show increasing trends, indicating that polarization is not saturated even at 200 V (rms). Although the AC poling effect did not reach saturation at 200 V, this limitation is attributed to the maximum output capacity of the current experimental apparatus. As higher AC voltages could not be applied, it remains possible that further enhancement in piezoelectric performance may be achieved using equipment capable of exceeding this voltage threshold. The number of domain walls may have increased, which contributed to the increase in the dielectric constant. It should be noted that in a previous study, *d*_33_ and the relative permittivity showed similar trends [22]. Based on the above, the applied voltage was set to 200 V (rms) in this experiment.

**Figure 10:**
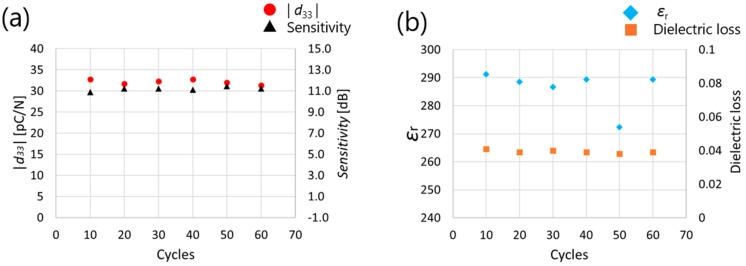
Measured (**a**) absolute value of *d*_33_ (**b**) relative dielectric constant results for AC poling (200 V (rms), 1 Hz) and as a function of the cycles on PZT/PZT.

Figure 10 shows the measurement results of *d*_33_, ultrasonic sensitivity, relative dielectric constant, and dielectric loss when polarization was performed by changing the number of applied cycles from 10 to 60. Cycles below 10 were not investigated, as previous studies [21,22] have shown that noticeable improvements in piezoelectric performance are generally not observed in such low-cycle regimes. Figure 10 shows that *d*_33_, ultrasonic sensitivity, and dielectric constant did not change between 10 and 60 cycles, indicating that the polarization was saturated at the 10-cycle stage. Therefore, the number of application cycles was set to 10 in the following experiments. The measured *d*_33_ value of the sol–gel composite film is lower than that of bulk sintered PZT and sol–gel-derived PZT thin films, as shown in Table 1. This reduction is primarily due to the porous nature of the composite material and the relatively low film thickness. However, the corresponding dielectric constant is also low, as shown in Figure 10b, which leads to a relatively high *g*_33_, enhancing the effectiveness of the material in ultrasonic sensing. As a result, despite the modest *d*_33_, the transducer exhibited sufficient sensitivity in pulse-echo measurements, confirming its applicability to phased-array ultrasonic systems.

**Figure 11:**
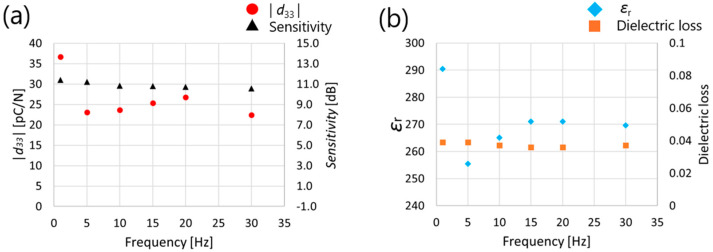
Measured (**a**) absolute value of *d*_33_ (**b**) relative dielectric constant results for AC poling (200 V (rms), 10 cycles) and as a function of the frequency on PZT/PZT.

Figure 11 shows the measurement results of *d*_33_, ultrasonic sensitivity, relative permittivity, and dielectric loss when polarization was performed by changing the frequency from 1 to 30 Hz. It was found that *d*_33_, ultrasonic sensitivity, and dielectric constant showed the highest values when an electrical field of 1 Hz was applied.

Based on the above-mentioned results, AC poling conditions with a voltage off-phase angle of 90°, 200 V (rms), 10 cycles, and 1 Hz were applied in the following experiments.

### 3.2. Comparison with DC Poling and Corona Discharge Poling

**Figure 12:**
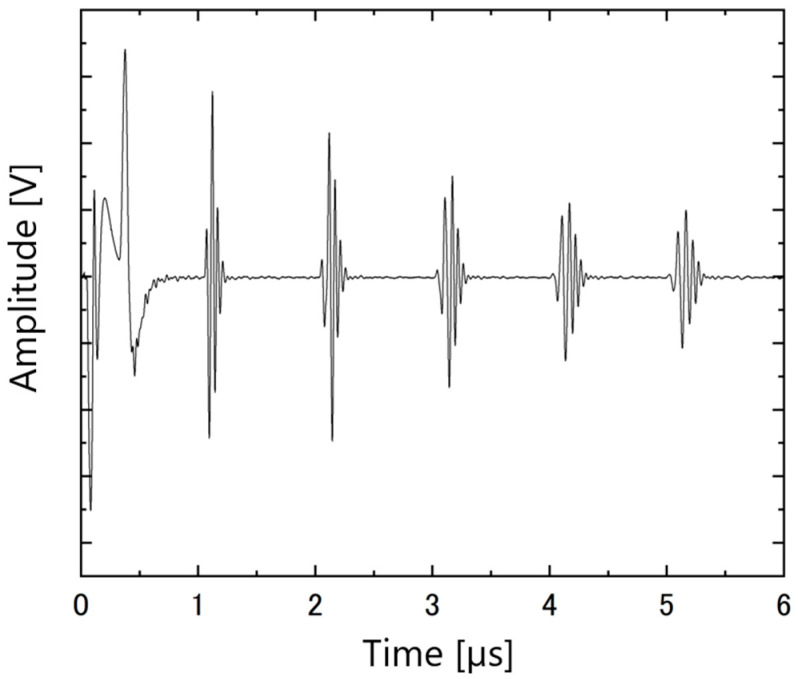
Ultrasonic response of PZT/PZT poled by AC on a titanium substrate with a thickness of 3 mm.

**Figure 13:**
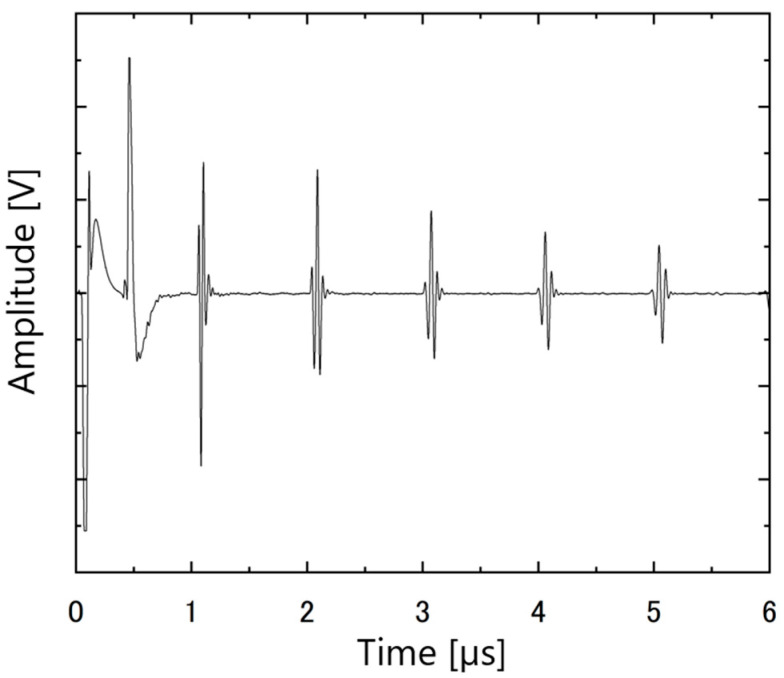
Ultrasonic response of PZT/PZT poled by corona on a titanium substrate with a thickness of 3 mm.

**Figure 14:**
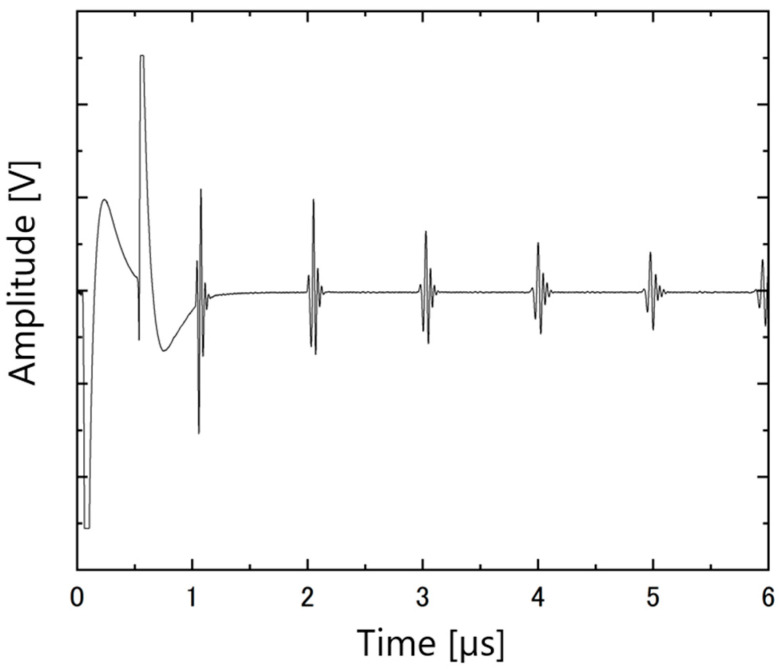
Ultrasonic response of PZT/PZT poled by DC on a titanium substrate with a thickness of 3 mm.

The ultrasonic responses of the PZT/PZT samples with AC poling, the corona discharge technique, and DC poling are shown in Figure 12, Figure 13 and Figure 14, respectively. In each figure, the arrival times of the reflected waves are reasonable based on the calculation of the traveling distance and sound velocity.

In this experiment, 2 × 2 electrodes simulating a matrix array were fabricated on a 20 mm × 20 mm piezoelectric film, as shown in Figure 3, and the uniformity of polarization for each poling technique was evaluated in terms of *d*_33_ and ultrasonic sensitivity dispersion. Table 3 shows the measured *d*_33_, ultrasonic sensitivity, and polarization time for each polarization technique used. The thickness of the piezoelectric film was confirmed to be uniform within ±1 μm across the surface, and this variation is considered negligible in terms of its effect on the poling characteristics.

Comparing the polarization time for each polarization, AC poling is superior in terms of polarization time because it requires only 10 s to complete, while DC poling requires 1200 s, and corona discharge polarization requires 300 s. In addition, the mean and variance of *d*_33_ and ultrasonic sensitivity of the AC-poled samples across the electrodes were −35.1 ± 0.8 pC/N and 11.4 ± 0.1 dB, whereas those of the DC-poled samples were −27.1 ± 0.7 pC/N and 2.5 ± 0.2 dB, and those poled by corona discharge showed values of −36.7 ± 2.9 pC/N and 7.2 ± 0.4 dB, respectively. These results indicate that the sample with AC poling has comparable *d*_33_ and ultrasonic sensitivity with corona discharge-poled samples and comparable dispersion values with DC poling samples, indicating that AC poling is a polarization method with excellent piezoelectric and ultrasonic properties and less variance. The lower *d*_33_ and ultrasonic sensitivity obtained by DC poling may be due to the generation of microcracks. Sparks were observed between the probe connected to the P/R and the top electrode during the ultrasonic measurements of the sample with the DC poling process. When microcracks exist in the film, the electric field locally increases, and sparks may be observed. The reduced piezoelectric response observed in the DC-poled samples is likely attributed to insufficient domain switching due to the limited duration (one minute) of the applied maximum electric field, as well as the possible formation of microcracks within the porous sol–gel composite structure. The large dispersion of *d*_33_ and ultrasonic sensitivity with corona discharge polarization is due to the non-uniformity of the plasma and film surface. In addition to the fact that corona discharges are known to exhibit nonlinear electric field distributions, the sol–gel composite is also likely to be strongly affected by the chaotic nature of corona discharge polarization because of the uneven membrane surface due to its porosity.

Because AC and DC poling have different current characteristics, it is expected that AC poling will suppress the dielectric breakdown. Assuming that the piezoelectric film is a series RC circuit for simplicity, the maximum value of the transient current when a DC voltage is applied appears at the instant of electrical field application. However, when an AC voltage is applied, the transient current is zero at the moment of electrical field application. Porous sol–gel composites have small *R* and *C*, and in this experiment, *f* is small (1–30 Hz). Consequently, AC poling is expected to have less dielectric breakdown and/or microcracks because the maximum transient current is suppressed compared with DC poling.

## 4. Conclusions

In this study, we attempted to apply AC poling to PZT/PZT sol–gel composites to overcome the non-uniformity of polarization caused by the corona discharge. AC poling was applied to a PZT/PZT sol–gel composite with a thickness of 25 µm fabricated on a titanium substrate, and its characteristics were studied. The highest electrical, piezoelectric, and ultrasonic properties were obtained under AC poling conditions with a voltage off-phase angle of 90°, an applied voltage of 200 V (rms), 10 application cycles, and a frequency of 1 Hz. The piezoelectric strain constant *d*_33_, ultrasonic sensitivity, and dispersion were evaluated for the AC, DC, and corona discharge poling techniques. The *d*_33_ and ultrasonic sensitivity for AC poling had an average value of −35.1 ± 0.8 pC/N and 11.4 ± 0.1 dB in dispersion, while the mean and variance of measured *d*_33_ and ultrasonic sensitivity for DC poling were −27.1 ± 0.7 pC/N and 2.5 ± 0.2 dB, respectively, and those for corona discharge polarization were −36.7 ± 2.9 pC/N and 7.2 ± 0.4 dB, respectively. These results indicate that AC polarization has *d*_33_ and ultrasonic sensitivity values comparable to those of corona discharge poling and dispersion values comparable to those of DC polarization. This indicates that AC poling is a polarization method that combines excellent piezoelectric and ultrasonic properties with uniform polarization.

## Figures and Tables

**Table 1:** Material properties of HIZIRCO L (PZT powder) and PZT sol–gel.

Material	$\varepsilon_{r}$	$d_{33}$ [pC/N]	$k_{33}$ [%]
HIZIRCO L	1800	400	70
PZT sol–gel [27]	~274	~21	-

**Table 2:** Voltage off-phase angle dependence of measured *d*_33_.

Voltage Off-Phase Angle [°]	Measured *d*_33_ [pC/N]
0	41.2
90	−33.5
180	−30.9
270	28.8

**Table 3:** Values of measured $d_{33}$, sensitivity, and poling time for each poling method.

Sample	Poling Method	Measured $d_{33}$ [pC/N]	Sensitivity [dB]	Poling Time [s]
PZT/PZT	AC	−35.1 ± 0.8	11.4 ± 0.1	10
	Corona	−36.7 ± 2.9	7.2 ± 0.4	300
	DC	−27.1± 0.7	2.5 ± 0.2	1200

## Data Availability

The raw data supporting the conclusions of this article will be made available by the authors on request.
